# Analysis of Factors Influencing the Clinical Severity of Omicron and Delta Variants

**DOI:** 10.3390/tropicalmed8060330

**Published:** 2023-06-20

**Authors:** Shanlu Zhao, Kaiwei Luo, Yichao Guo, Mingli Fang, Qianlai Sun, Zhihui Dai, Hao Yang, Zhifei Zhan, Shixiong Hu, Tianmu Chen, Xiaojun Li

**Affiliations:** 1 Hunan Provincial Center for Disease Control and Prevention (Workstation for Emerging Infectious Disease Control and Prevention, Chinese Academy of Medical Sciences), Changsha 410005, China; 2 School of Public Health, Xiamen University, Xiamen 361102, China

**Keywords:** COVID-19, SARS-Cov-2, vaccination, clinical severity

## Abstract

The Omicron variant is the dominant strain circulating globally, and studies have shown that Omicron cases have milder symptoms than Delta cases. This study aimed to analyze the factors that affect the clinical severity of Omicron and Delta variants, evaluate and compare the effectiveness of COVID-19 vaccines with different technological platforms, and assess the vaccine effectiveness against different variants. We retrospectively collected the basic information of all local COVID-19 cases reported by Hunan Province to the National Notifiable Infectious Disease Reporting System from January 2021 to February 2023, including gender, age, clinical severity, and COVID-19 vaccination history. From 1 January 2021 to 28 February 2023, Hunan Province reported a total of 60,668 local COVID-19 cases, of which, 134 were infected with the Delta variant and 60,534 were infected with the Omicron variant. The results showed that infection with the Omicron variant (adjusted OR (aOR): 0.21, 95% CI: 0.14–0.31), getting vaccinated (booster immunization vs. unvaccinated aOR: 0.30, 95% CI: 0.23–0.39) and being female (aOR: 0.82, 95% CI: 0.79–0.85) were protective factors for pneumonia, while old age (≥60 years vs. <3 years aOR: 4.58, 95% CI: 3.36–6.22) was a risk factor for pneumonia. Being vaccinated (booster immunization vs. unvaccinated aOR: 0.11, 95% CI: 0.09–0.15) and female (aOR: 0.54, 95% CI: 0.50–0.59) were protective factors for severe cases, while older age (≥60 years vs. < 3 years aOR: 4.95, 95% CI: 1.83–13.39) was a risk factor for severe cases. The three types of vaccines had protective effects on both pneumonia and severe cases, and the protective effect on severe cases was better than that on pneumonia. The recombinant subunit vaccine booster immunization had the best protective effect on pneumonia and severe cases, with ORs of 0.29 (95% CI: 0.2–0.44) and 0.06 (95% CI: 0.02–0.17), respectively. The risk of pneumonia from Omicron variant infection was lower than that from Delta. Chinese-produced vaccines had protective effects on both pneumonia and severe cases, with recombinant subunit vaccines having the best protective effect on pneumonia and severe pneumonia cases. Booster immunization should be advocated in COVID-19 pandemic-related control and prevention policies, especially for the elderly, and booster immunization should be accelerated.

## 1. Introduction

During the COVID-19 pandemic, multiple variants emerged that differed in their transmissibility and in the clinical severity they caused. For example, compared with Delta, the risk of severe outcomes after infection with Omicron variant is much lower [1]. Studies from the United States, Denmark, the United Kingdom, and Qatar have shown that Omicron variant infection may be associated with a lower risk of hospitalization and milder clinical outcomes compared with the Delta variant [1,2,3,4]. While COVID-19 vaccines created using various technical routes have good protective effects against serious disease and mortality, their performance in avoiding COVID-19 infection is not optimal [5,6,7]. A meta-analysis study evaluating the real-world vaccine effectiveness showed that the overall incidence of variants after the first and second vaccines was 0.07 and 0.03, respectively. After the second vaccination, the VE of the occurrence of variants between the vaccine and the placebo or unvaccinated group was 40% and 96%, respectively [8]. On 26 November 2021, the Omicron variant (B.1.1.529) was declared a variant of concern by the World Health Organization (WHO) [9]. The Omicron variant was first detected in South Africa in November 2021, but quickly spread globally and is now the dominant variant in most countries [10,11] and the main variant in China [12]. In early 2022, Hunan reported the first case of Omicron, and in April 2022, cluster outbreaks of Omicron occurred in Huaihua and Shaoyang cities of Hunan province. Since October 2022, Omicron has spread rapidly within Hunan province, reaching a peak of incidence in December. Studies have shown that most Omicron cases have occurred through breakthrough infections, but vaccination still effectively reduces the incidence of severe cases [13]. China started mass vaccination against COVID-19 on 15 December 2020 [14]. In the three years of COVID-19 prevention and control, Hunan province has been vigorously promoting COVID-19 vaccination, and the types of COVID-19 vaccines administered in Hunan province include three types: inactivated vaccine, recombinant subunit vaccine, and adenovirus vector vaccine. Domestic and foreign studies have shown that COVID-19 vaccines of different technical routes have good protective effects against severe and death. A real-world study from South Africa showed that mRNA COVID-19 vaccine provides 93% protection against the Delta variant 14 days after vaccination, while it drops to 70% against the Omicron strain [15]. The main COVID-19 vaccine administered in mainland China is the inactivated vaccine, and the current domestic real-world studies are also mainly for the inactivated vaccine. A real-world study in the Guangdong province of China showed that two doses of the inactivated vaccine had a protective effect of 59.0% against the COVID-19 Delta variant and a protective effect of 70.2% against severe COVID-19 [16]. A study in the Jilin province of China showed that two and three doses of inactivated vaccine could reduce the risk of pneumonia and severe cases caused by Omicron variant by 71% and 74%, respectively [17]. There are less real-world data on China’s recombinant subunit vaccine and adenovirus vector vaccine, and there is also a lack of comparison of the real-world effectiveness of different types of COVID-19 vaccines. Therefore, this study aimed to analyze the factors affecting the clinical severity of Omicron and Delta variants, to evaluate and compare the effectiveness of different technical routes of COVID-19 vaccines, and to assess the protective effect of vaccination history on different variants.

## 2. Materials and Methods

### 2.1. Data Sources

This study is a retrospective analysis. The data source was the National Notifiable Infectious Disease Reporting System. All local COVID-19 cases (including asymptomatic infections) reported in Hunan Province from January 2021 to February 2023 were included in the analysis. The individual COVID-19 vaccine immunization history information in the National Immunization Program Information System was matched by the case ID number.

### 2.2. Vaccination and Clinical Outcome Definition

The immunization history of this study was divided into four groups: unvaccinated, partially vaccinated, fully vaccinated, and booster immunization. Unvaccinated means that no COVID-19 vaccine was administered before onset or on the day of onset; partially vaccinated means that primary immunization was not completed before onset or full basic immunization was completed <14 days; fully vaccinated means that primary immunization was completed before onset (according to the immunization procedure, 2 doses of inactivated vaccine, 1 dose of adenovirus vector vaccine, or 3 doses of recombinant subunit vaccine were administered) ≥14 days; booster immunization means that ≥7 days after completing primary immunization, an additional dose was administered. 

The clinical classification diagnosis of COVID-19 cases was based on the “National Protocols for COVID-19 Prevention and Control” (Eighth Edition Revised Version), (Ninth Edition), (Tenth Edition) [18,19,20]. According to the most severe clinical outcomes recorded in the data source, the cases in this study were divided into (1) pneumonia cases, including mild and moderate cases; (2) severe cases, including severe and critical cases. The asymptomatic cases mean that no symptoms were reported to authorities at the time of contact investigation. Once they developed symptoms, they would have been categorized as confirmed symptomatic cases. 

### 2.3. Statistical Analysis

The following analyses were performed on the data using SPSS17.0 software: (1) Descriptive analysis of the age, vaccination status, and gender distribution of COVID-19 cases with different variants and clinical outcomes. (2) Logistic regression analysis was used to analyze the influencing factors of COVID-19 pneumonia and severe illness occurrence, as well as the impact of different technical route vaccines on clinical outcomes. (3) Univariate logistic regression analysis and exact logistic regression analysis were performed on pneumonia cases and severe cases of COVID-19 with different variants and age groups ≥3 years old, respectively. The odds ratio (OR) and its 95% confidence interval (CI) of different immunization histories were calculated separately. The risk reduction of COVID-19 pneumonia and severe illness was calculated by (1−OR) × 100%. *p* < 0.05 was considered statistically significant. The data were analyzed by IBM SPSS Statistics for Windows, Version 21.0. Armonk, NY, USA: IBM Corp.

## 3. Results

### 3.1. Epidemiology Characteristics

In total, 60,668 cases of local COVID-19 cases reported in Hunan Province from 1 January 2021 to 28 February 2023, were included in this study. Among them, 134 were infected with the Delta variant, and 60,534 were infected with the Omicron variant. Among the 134 cases of the Delta variant infection, 20 (14.9%) were asymptomatic; among the 60,534 cases of the Omicron variant infection, 9188 (15.2%) were asymptomatic. The difference in the proportion of asymptomatic infections between the Delta and Omicron variants was not statistically significant (χ 2= 0.007, *p* = 0.935). Among the COVID-19 cases infected with the Delta variant, 44.8% (60 cases) had no vaccination history, 53.7% (72 cases) were female, 8.2% (11 cases) were aged ≥60 years, 89 cases developed pneumonia, and 6 cases were severe cases. Among the cases infected with the Omicron variant, 52.6% (31,854 cases) completed booster immunization, 52.6% (31,821 cases) were male, 61.3% (37,110 cases) were aged ≥60 years, 23,107 cases developed pneumonia, and 2617 cases were severe cases (Table 1).

### 3.2. Logistic Regression Analyses

Multiple logistic regression analysis was performed with the clinical outcome as the dependent variable. The results showed that infection with the Omicron variant (OR = 0.21, 95% CI: 0.14~0.31), COVID-19 vaccination and being female (OR = 0.82, 95% CI: 0.79~0.85) were protective factors for developing pneumonia, while older age was a risk factor. Compared with the <3-years-old age group, the 3–17 year age group had a pneumonia risk that was 0.4 times lower (OR = 0.40, 95% CI: 0.29~0.55), while the 18–59 year and ≥60 year age groups had pneumonia risks that were 1.67 (95% CI: 1.23~2.28) and 4.58 (95% CI: 3.36~6.22) times higher, respectively. COVID-19 vaccination and female sex (OR = 0.54, 95% CI: 0.5–0.59) were protective factors for developing severe illness, while older age was a risk factor (Table 2). Compared with those who were unvaccinated, partial vaccination had an OR of 0.26 (95% CI: 0.19–0.34), full vaccination had an OR of 0.16 (95% CI: 0.12–0.22), and booster vaccination had an OR of 0.11 (95% CI: 0.09–0.15) against severe COVID-19. 

A logistic regression analysis was performed with variant, vaccination history, sex, and age as independent variables, and the clinical outcomes of cases vaccinated with inactivated vaccines, adenovirus vector vaccines, and recombinant subunit vaccines as dependent variables. The results showed that all three types of vaccines had a protective effect against pneumonia and severe illness, with better protection against severe symptoms than pneumonia. 

Compared with unvaccinated cases, the risk of developing pneumonia for partially vaccinated, fully vaccinated, and those who received a booster dose of inactivated vaccines were 0.53 (95% CI: 0.41–0.69), 0.37 (95% CI: 0.29–0.48), and 0.31 (95% CI: 0.24–0.39), respectively. The risk of developing severe illness for partially vaccinated, fully vaccinated, and those who received a booster dose of inactivated vaccines were 0.26 (95% CI: 0.19–0.36), 0.19 (95% CI: 0.14–0.25), and 0.11 (95% CI: 0.09–0.15), respectively. The risks of developing pneumonia among those who received partial, full, and booster doses of inactivated virus vector vaccines were 0.99 (95% CI: 0.3~3.27), 0.48 (95% CI: 0.35~0.65), and 0.36 (95% CI: 0.24~0.52), respectively. The risks of developing severe disease were 0.37 (95% CI: 0.08~1.7), 0.28 (95% CI: 0.19~0.41), and 0.16 (95% CI: 0.09~0.29), respectively. For those who received partial, full, and booster doses of recombinant subunit vaccines, the risks of developing pneumonia were 0.49 (95% CI: 0.37~6.5), 0.34 (95% CI: 0.26~0.44), and 0.29 (95% CI: 0.2~0.44), respectively. The risks of developing severe disease were 0.26 (95% CI: 0.19~0.35), 0.14 (95% CI: 0.11~0.19), and 0.06 (95% CI: 0.02~0.17), respectively. The booster immunization with the recombinant subunit vaccine had the best protective effect against pneumonia and severe symptoms, with ORs of 0.29 (95% CI, 0.2–0.44) and 0.06 (95% CI, 0.02–0.17), respectively (Table 3).

Using gender as a confounding factor, the analysis was stratified based on different variants, age groups, and vaccination histories. Among Delta variant cases, there was no statistically significant difference in the odds ratio of pneumonia and severe illness among cases with different vaccination histories in each age group. Among Omicron variant cases, compared with unvaccinated cases in the same age group, both full vaccination and booster immunization had statistically significant OR values for reducing the risk of pneumonia in the 18–59 years and ≥60 years age groups. Fully vaccination reduced the risk of developing pneumonia by 74% (OR = 0.26, 95% CI: 0.13–0.56) and 62% (OR = 0.38, 95% CI: 0.28–0.50) in the 18–59 years and ≥60 years age groups, respectively, and reduced the risk of severe illness by 98% (OR = 0.02, 95% CI: 0.01–0.04) and 78% (OR = 0.22, 95% CI: 0.16–0.29) in the 18–59 years and ≥60 years age groups, respectively. Similarly, booster immunization reduced the risk of pneumonia by 77% (OR = 0.23, 95% CI: 0.11–0.48) and 70% (OR = 0.30, 95% CI: 0.23–0.40) in the 18–59 years and ≥60 years age groups, respectively, and reduced the risk of severe illness by 99% (OR = 0.01, 95% CI: 0.01–0.03) and 85% (OR = 0.15, 95% CI: 0.11–0.20) in the 18–59 years and ≥60 years age groups, respectively (Table 4).

## 4. Discussion

This study found that the proportion of asymptomatic infections for the Delta and Omicron variants were 14.9% and 15.2%, respectively, with no statistically significant difference. According to research conducted by Peking University, the variant is one of the factors influencing the proportion of asymptomatic cases in COVID-19 patients, and the proportion of asymptomatic infections during the Omicron epidemic was higher than that of previous variants. In 2022, the proportion of asymptomatic cases was 33.72%, while in 2021 it was 23.57% [21]. Our study did not observe such a statistical difference, which may be due to different investigation periods and variations in the populations’ immune levels. The study also found that the risk of developing pneumonia from the Omicron variant was 0.21 times that of Delta, with an OR of 0.21 (95% CI: 0.14–0.31). This result is consistent with findings from studies conducted in the US, Denmark, the UK, and South Africa, indicating that Omicron is associated with lower clinical severity compared to Delta [1,2,3,22]. However, it should be noted that even though Omicron may cause less severe disease, its high transmissibility and a large number of infections could further burden the healthcare system, especially in developing countries [23,24,25]. In the Hunan province of China, there were 23,361 cases of pneumonia caused by Omicron (89 cases by Delta), and 2640 cases of severe illness (6 cases by Delta).

Compared to females, males were at higher risk of developing pneumonia and severe illness after being infected with COVID-19. This is similar to previous research results [26,27]. A meta-analysis of 3,111,714 COVID-19 patients worldwide showed that men infected with COVID-19 were three times more likely to require intensive care and had a significantly higher risk of death compared to women [28]. This may be due to the fact that female patients have a stronger T-cell immune response during COVID-19 infection [29], or it may be related to genetic factors and sex hormones [30].

Even after adjusting for variant and vaccination history, age was strongly associated with the occurrence of pneumonia and severe illness. This study found that among those under 18 years old, the risk of pneumonia and severe illness was 0.4 and 0.23 times that of those under 3 years old, respectively. This result is similar to studies conducted in the United States and Europe [31,32,33]. Given that the immune system of infants and young children is not yet mature, some children may develop multisystem inflammatory syndrome [31,32,33], along with the susceptibility of young children to severe illness caused by the influenza virus [34]. In addition, compared to children and adolescents, adults infected with COVID-19 tend to experience more severe symptoms. Our study showed that the risk of pneumonia in the 18–59 and ≥60 age groups was 1.67 and 4.58 times that of those under 3 years old, respectively, while the risk of severe illness was 1.23 and 4.95 times higher, respectively. These findings suggest that the risk of pneumonia and severe illness increases with age in adults, with the ≥60 age group having the highest risk. As is well known, age is one of the risk factors for poor prognosis in SARS-CoV-2-infected patients. According to data from 79,394 COVID-19 patients in mainland China, the likelihood of death after symptom onset for patients over 59 years old was 5.1 times higher (95% CI: 4.2–6.1) than that of patients aged 30–59 [35]. A meta-analysis including 212 studies from 11 countries or regions found that 60.8% (95% CI: 57.2–64.2) of severe COVID-19 patients were male, with an average age of 60.4 years old [36]. The elderly usually have underlying diseases, weakened physical condition, aging of the immune system, and higher SARS-CoV-2 viral loads, which may increase their risk of adverse outcomes and higher mortality when infected with COVID-19 [37].

Compared to the abundant research data on mRNA vaccines and adenovirus vector vaccines for COVID-19, there are relatively few real-world estimations available for Chinese-made COVID-19 vaccines. A cohort study from Denmark, where mainly mRNA vaccines are administered, found that the risk of hospitalization was reduced by 29% and 50% after receiving two doses and three doses of vaccine, respectively [3]. This study found that compared with unvaccinated individuals, the risks of developing pneumonia could be reduced by 48%, 63%, and 70% after partial vaccination, full vaccination, and booster immunization, respectively. Similarly, the risks of developing severe illness could be reduced by 74%, 84%, and 89%, respectively. These findings suggest that vaccines protect against pneumonia and severe illness. A real-world study from Jilin Province, China, showed that Chinese-made inactivated vaccines protect against pneumonia and severe illness caused by the Omicron variant, which booster vaccination can further enhance [17]. Compared with unvaccinated individuals, receiving two doses or three doses of Chinese-made inactivated vaccine can reduce the risks of developing pneumonia or severe illness by 60% and 68%, respectively, which is consistent with the results of this study. Additionally, this study is consistent with another study indicating that the effect of booster immunization in reducing the risk of developing pneumonia is better [5]. The effect may be due to the booster dose’s restoration of the neutralizing antibody activity, which significantly increases its protective effect [38].

All three Chinese-produced vaccines have sound protective effects against pneumonia and severe illness. Full vaccination with inactivated vaccines reduces the risk of COVID-19 and severe disease by 63% and 81%, respectively. Full vaccination with adeno-vector vaccines reduces the risk of pneumonia and severe disease by 52% and 72%, respectively, while full vaccination with recombinant subunit vaccines reduces the risk of pneumonia and severe disease by 66% and 86%, respectively. Booster immunization further reduces the risk of pneumonia and severe disease by 69% and 89% for inactivated vaccines, 64% and 84% for adeno-vector vaccines, and 71% and 94% for recombinant subunit vaccines. We observed that the recombinant subunit vaccine, whether for a full vaccination regimen or booster immunization, had the best risk reduction for pneumonia and severe illness, particularly with the risk of severe illness being reduced by 86% and 94%, respectively. Additionally, real-world data from abroad also showed that recombinant subunit vaccines can significantly reduce hospitalization rates, as well as severe illness and death caused by COVID-19 [6,39]. Clinical data from a phase three trial of the recombinant subunit vaccine showed an efficacy of 87.6% (95% CI: 70.6–95.7) in preventing severe to critical COVID-19 [39]. mRNA vaccines have shown good efficacy, possibly due to their ability to induce higher titers of neutralizing antibodies and better cellular immunity. They may also induce and enrich neutralizing antibodies directed towards the receptor-binding domain (RBD) of the virus, which have resistance against SARS-CoV-2 mutations due to their ability to bind to multiple RBD binding patterns [40,41]. As Dr. Hanna Nohynek of the World Health Organization said, we need to accelerate the development of new and efficient vaccines that can reduce mild infections and virus transmission to reduce the impact of the new wave of the pandemic [37].

In addition, according to our results, the risk of developing pneumonia or severe illness after vaccination varies among different age groups. In the age group of ≥60 years, the full regimen of Chinese-produced vaccines can reduce the risk of pneumonia caused by the Omicron variant by 62%, and the booster dose can reduce the risk of severe illness caused by the Omicron variant in the ≥60 age group by 85%. This is consistent with a national study in China [14], which found that fully vaccinated individuals aged 60 years or older had a 65% reduction in the risk of developing pneumonia after infection with the Omicron variant, and those who received a booster shot had a 90% reduction in the risk of developing severe illness. A large-scale study in France involving approximately 22 million people showed that COVID-19 vaccination could reduce the hospitalization risk of people aged 75 and above by 86% [42]. These results suggest that COVID-19 vaccination can significantly reduce the risk of pneumonia and severe illness in older adults, and it is recommended to implement full vaccination and booster immunization for eligible elderly individuals as soon as possible. Elderly people remain a high-risk group for severe illness and death from COVID-19, and the older the age, the higher the risk of severe illness and death. Multiple studies on the optimal vaccination strategy for COVID-19 have shown that to reduce the severity of the disease and the subsequent deaths, vaccination should prioritize the elderly population. A study in the United States showed that if the goal is to minimize the number of deaths, it is best to first distribute the vaccine to the high-risk (older) age group [43]. A model study in the UK suggested that prioritizing vaccination for the elderly is the most effective strategy to date for reducing subsequent deaths [44]. A model study in Wuhan, China also indicated that to reduce the severity of COVID-19 disease, vaccination should prioritize the elderly population [45].

This study has the following limitations. Firstly, there were fewer cases of Delta variant infections, and the sample size for stratified analysis was insufficient, which may have led to bias in the results. Secondly, this study mainly analyzed the effects of vaccine immunity, age group, and gender on clinical outcomes and did not consider the influence of underlying diseases on the cases. Thirdly, during the Omicron variant epidemic, there may have been cases that were not detected or not treated, so the risk of Omicron infection causing pneumonia and hospitalization may be underestimated. Finally, this study is based on a case-only analysis conducted on COVID-19 cases, and it does not include the uninfected population. This limitation in the selection of the control group may not fully reflect the protective effects of the vaccines.

## 5. Conclusions

Our study suggests that the risk of pneumonia from Omicron variant infection is lower than that of the Delta variant. Chinese-produced vaccines have good protective effects against pneumonia and severe cases, and the recombinant subunit vaccine has the best protective effect against pneumonia and severe cases. It is recommended to strengthen immunization in the control and prevention policies related to the COVID-19 pandemic, especially for the elderly, who should be prioritized for the implementation of the booster vaccination.

## Figures and Tables

**Table 1:** Characteristics of 60,668 COVID-19 cases in Hunan Province from January 2021 to February 2023.

Factors	COVID-19 Infection		Pneumonia		Severe Illness	
	Omicron (%)	Delta (%)	Omicron (%)	Delta (%)	Omicron (%)	Delta (%)
Vaccination history						
Unvaccinated	273 (0.5)	60 (44.8)	192 (0.8)	37 (41.6)	75 (2.9)	5 (83.3)
Partially	4813 (8.0)	31 (23.1)	2572 (11.1)	25 (28.1)	423 (16.2)	0 (0)
Fully	23,594 (39.0)	43 (32.1)	8506 (36.8)	27 (30.3)	999 (38.2)	1 (16.7)
Booster	31,854 (52.6)	0 (0)	11,837 (51.2)	0 (0)	1120 (42.8)	0 (0)
Sex						
Male	31,821 (52.6)	62 (46.3)	13,144 (56.9)	39 (43.8)	1798 (68.7)	2 (33.3)
Female	28,713 (47.4)	72 (53.7)	9963 (43.1)	50 (56.2)	819 (31.3)	4 (66.7)
Age group						
<3	288 (0.5)	0 (0)	50 (0.2)	0 (0)	4 (0.2)	0 (0)
3–17	4432 (7.3)	38 (28.4)	363 (1.6)	19 (21.3)	16 (0.6)	0 (0)
18–59	18,704 (30.9)	85 (63.4)	4608 (19.9)	62 (69.7)	261 (10)	4 (66.7)
≥60	37,110 (61.3)	11 (8.2)	18,086 (78.3)	8 (9.0)	2336 (89.3)	2 (33.3)
Total	60,534 (100)	134 (100)	23,107 (100)	89 (100)	2617 (100)	6 (100)

**Table 2:** Odds ratios of factors influencing the clinical outcomes of COVID-19 cases.

Factors	Case Number	Pneumonia			Severe Illness		
		*n* (%)	*p*	OR (95% CI)	*n* (%)	*p*	OR (95% CI)
Variant							
Delta	134	89 (66.4)		1 (Ref)	6(4.5)		1 (Ref)
Omicron	60,534	23,107 (38.2)	<0.0001	0.21 (0.14–0.31)	2617(4.3)	0.911	1.05 (0.43–2.57)
Vaccination history			<0.0001			<0.0001	
Unvaccinated	333	229 (68.8)		1 (Ref)	80 (24)		1 (Ref)
Partially	4844	2597 (53.6)	<0.0001	0.52 (0.40–0.67)	423 (8.7)	<0.0001	0.26 (0.19–0.34)
Fully	23,637	8533 (36.1)	<0.0001	0.37 (0.29–0.47)	1000 (4.2)	<0.0001	0.16 (0.12–0.22)
Booster	31,854	11,837 (37.2)	<0.0001	0.30 (0.23–0.39)	1120 (3.5)	<0.0001	0.11 (0.09–0.15)
Sex							
Male	31,883	13,183 (41.3)		1 (Ref)	1800 (5.6)		1 (Ref)
Female	28,785	10,013 (34.8)	<0.0001	0.82 (0.79–0.85)	823 (2.9)	<0.0001	0.54 (0.5–0.59)
Age group			<0.0001				
<3	288	50 (17.4)		1 (Ref)	4 (1.4)		1 (Ref)
3–17	4470	382 (8.5)	<0.0001	0.4 (0.29–0.55)	16 (0.4)	0.009	0.23 (0.08–0.69)
18–59	18,789	4670 (24.9)	0.001	1.67 (1.23–2.28)	265 (1.4)	0.689	1.23 (0.45–3.34)
≥60	37,121	18,094 (48.7)	<0.0001	4.58 (3.36–6.22)	2338 (6.3)	0.002	4.95 (1.83–13.39)

**Table 3:** Regression analysis of different COVID-19 vaccine technology routes on the clinical outcomes.

Vaccine Type	Vaccination History	Case Number	Pneumonia			Severe Illness		
			*n* (%)	*p*	OR (95% CI)	*n* (%)	*p*	OR (95% CI)
Inactivated vaccine	Vaccination history			<0.0001			<0.0001	
	Unvaccinated	333	229 (68.8)		1 (Ref)	80 (24.0)		1 (Ref)
	Partially	2126	1094 (51.5)	<0.0001	0.53 (0.41–0.69)	177 (8.3)	<0.0001	0.26 (0.19–0.36)
	Fully	11,274	3324 (29.5)	<0.0001	0.37 (0.29–0.48)	425 (3.8)	<0.0001	0.19 (0.14–0.25)
	Booster	31,436	11,660 (37.1)	<0.0001	0.31 (0.24–0.39)	1102 (3.5)	<0.0001	0.11 (0.09–0.15)
Adenovirus vector vaccine	Vaccination history			<0.0001			<0.0001	
	Unvaccinated	333	229 (68.8)		1 (Ref)	80 (24)		1 (Ref)
	Partially	15	11 (73.3)	0.99	0.99 (0.3–3.27)	2 (13.3)	0.201	0.37 (0.08–1.7)
	Fully	608	346 (56.9)	<0.0001	0.48 (0.35–0.65)	58 (9.5)	<0.0001	0.28 (0.19–0.41)
	Booster	249	106 (42.6)	<0.0001	0.36 (0.24–0.52)	14 (5.6)	<0.0001	0.16 (0.09–0.29)
Recombinant subunit vaccine	Vaccination history			<0.0001			<0.0001	
	Unvaccinated	333	229 (68.8)		1 (Ref)	80 (24)		1 (Ref)
	Partially	2703	1492 (55.2)	<0.0001	0.49 (0.37–0.65)	244 (9)	<0.0001	0.26 (0.19–0.35)
	Fully	11,755	4863 (41.4)	<0.0001	0.34 (0.26–0.44)	517 (4.4)	<0.0001	0.14 (0.11–0.19)
	Booster	169	71 (42)	<0.0001	0.29 (0.2–0.44)	4 (2.4)	<0.0001	0.06 (0.02–0.17)

**Table 4:** The impact of different vaccination histories on clinical outcomes.

Variant	Age Group	Vaccination History	Case Number	Pneumonia			Severe Illness		
				*n* (%)	*p*	OR (95% CI)	*n* (%)	*p*	OR (95% CI)
Delta	3–17	Unvaccinated	38	19 (50.0)		Ref	0 (0)		Ref
		Partially	0	0 (0)	-	-	0 (0)	-	-
		Fully	0	0 (0)	-	-	0 (0)	-	-
		Total	38	19 (50.0)			0 (0)		
	18–59	Unvaccinated	13	11 (84.6)	0.208	Ref	3 (23.1)	0.113	Ref
		Partially	30	30 (100)	0.727	0.73 (0.13–4.24)	0 (0)	0.998	0
		Fully	42	21 (50.0)	0.181	0.33 (0.06–1.69)	1 (2.4)	0.037	0.08 (0.01–0.85)
		Total	85	62 (72.9)			4 (4.7)		
	≥60	Unvaccinated	9	7 (87.5)	1	Ref	2 (22.2)	1	Ref
		Partially	1	1 (100)	1	-	0 (0)	1	-
		Fully	1	0 (0)	1	-	0 (0)	1	-
		Total	11	8 (72.7)			2 (18.2)		
Omicron	3–17	Unvaccinated	2	0 (0)	0.66	Ref	0 (0)	1	Ref
		Partially	134	15 (4.1)	0.999	-	0 (0)	1	-
		Fully	4202	340 (93.7)	0.999	-	16 (100)	1	-
		Booster	94	8 (2.2)	0.999	-	0 (0)	1	-
		Total	4432	361 (8.1)			16 (0.4)		
	18–59	Unvaccinated	28	16 (0.3)	<0.0001	Ref	12 (4.6)	<0.0001	Ref
		Partially	746	244 (5.3)	0.009	0.36 (0.17–0.78)	29 (11.1)	<0.0001	0.05 (0.02–0.11)
		Fully	6608	1721 (37.3)	0.001	0.26 (0.13–0.56)	99 (37.9)	<0.0001	0.02 (0.01–0.04)
		Booster	11,322	2627 (57)	<0.0001	0.23 (0.11–0.48)	121 (46.4)	<0.0001	0.01 (0.01–0.03)
		Total	18,704	4608 (24.6)			261 (1.4)		
	≥60	Unvaccinated	235	172 (1)	<0.0001	Ref	60 (2.6)	<0.0001	Ref
		Partially	3913	2309 (12.8)	<0.0001	0.53 (0.39–0.71)	394 (16.9)	<0.0001	0.33 (0.24–0.45)
		Fully	12,667	6427 (35.5)	<0.0001	0.38 (0.28–0.5)	883 (37.8)	<0.0001	0.22 (0.16–0.29)
		Booster	20,295	9178 (50.7)	<0.0001	0.3 (0.23–0.4)	999 (42.8)	<0.0001	0.15 (0.11–0.2)
		Total	37,110	18,086 (48.7)			2336 (6.3)		

## Data Availability

Data cannot be shared publicly because they are confidential. Data are available from the respective department of Hunan CDC for researchers who meet the criteria for access to confidential data.
